# Dental anomalies as a possible clue of 1p36 deletion syndrome due to germline mosaicism: a case report

**DOI:** 10.1186/s12887-020-02049-1

**Published:** 2020-05-09

**Authors:** D. Nistico’, F. Guidolin, C. O. Navarra, M. Bobbo, A. Magnolato, A. P. D’Adamo, E. Giorgio, B. Pivetta, E. Barbi, P. Gasparini, M. Cadenaro, F. Sirchia

**Affiliations:** 1 University of Trieste, Trieste, Italy; 2 University of Padua, Padua, Italy; 3 Institute for Maternal and Child Health IRCCS Burlo Garofolo, Trieste, Italy; 4 Department Medical Sciences, University of Torino, Torino, Italy; 5 Division of Medical Genetics, AAS n.5 Friuli Occidentale, Pordenone, Italy

**Keywords:** 1p36 deletion syndrome, Monosomy 1p36 syndrome, Germline mosaicism, Dental anomalies, Recurrent microdeletion, Intellectual disability, Case report

## Abstract

**Background:**

Monosomy 1p36 is the most common terminal deletion syndrome with an autosomal dominant pattern of inheritance. This syndrome is defined by an extremely wide spectrum of characteristics; however, developmental delay and intellectual disability of various degree are present in all patients and about the 90% of patients have a severe intellectual disability. Dental agenesis or other dental anomalies have not been described in previous reports.

**Case presentation:**

We report the case of two little sisters born from healthy and non-consanguineous parents, presenting with dental anomalies and one of them with epilepsy, dilated cardiomyopathy with left-ventricular non-compaction, strabismus, history of poor growth, hypotonia and mild language delay. Patients were evaluated in several departments (genetic, child neuropsychiatric, cardiology, odontostomatology, ophthalmology, otorhinolaryngology) of Institute for Maternal and Child Health, IRCCS Burlo Garofolo, Trieste, Italy. They underwent investigations such as electrocardiogram, echocardiogram, dental orthopantomography X-Ray and Computed Tomography, electroencephalograms, abdomen ultrasound, blood tests, IQ tests, genetic analysis. They both have an Intelligence Quotient greater than 70 and a negative neurologic exam. Each sister carries the same 1p36 deletion of about 2.3 Mb. Genetic analysis of the parents’ blood samples (Single Nucleotide Polymorphism- array, karyotype and Fluorescent In Situ Hybridization) did not reveal any deletion, translocation or inversion and confirmed the paternity. A third sib of the probands does not carry the 1p36 deletion or other quantitative alterations.

**Conclusion:**

This report describes a new trait linked to monosomy 1p36, namely a mild intellectual outcome associated with significant dental anomalies. Our finding suggests that 1p36 deletion syndrome may present with a mild cognitive impairment or even with a normal intellectual development: this is very important for the genetic counselling, especially in a prenatal setting. Moreover, we report the third study with recurrent 1p36 deletion syndrome in two siblings, likely due to germline mosaicism. Finally, we believe that the dental anomalies should be investigated in 1p36 deletion syndrome and that the spectrum of the condition could be broader than we assume.

## Background

Monosomy 1p36 is the most common terminal chromosomal deletion in humans [1] and one of the most common microdeletion syndromes [2], with a prevalence of 1:5000–1:10,000 [3]. Though characterized by autosomal dominant transmission, 52% of the cases are de-novo terminal deletions, 29% are de-novo interstitial deletions, 12% are complex chromosome rearrangements (e.g. more than one 1p36 deletion or a 1p36 deletion with a 1p36 duplication), 7% are derivative chromosome 1 (e.g. 1p telomeric region is replaced by another chromosome end) [4]. Two examples of 1p36 deletion syndrome in siblings due to germline mosaicism have been described in literature [2, 5].

A precise genotype-phenotype correlation and the determination of the genes responsible for specific phenotypic features are not still certainty established [6]. 1p36 deletion syndrome is characterized by an extremely wide spectrum of features including intellectual disability, hypotonia, structural brain abnormalities including white matter anomalies (e.g. periventricular leukomalacia, hypoxic ischemic encephalopathy-like phenotype) [7], epilepsy, behavior disorder, hearing loss, ophthalmologic abnormalities, short stature, congenital heart defects (Ebstein’s anomaly, valvular anomalies, tetralogy of Fallot, ventricular septal defects), cardiomyopathy, genitourinary malformations, skeletal anomalies and variable dysmorphisms [1–3, 5, 8]. Developmental delay and intellectual disability of various degree are present in all patients and are hallmarks of the syndrome. Only 10% of patients with 1p36 deletion show mild to moderate cognitive impairment, while the remaining 90% have severe intellectual disability [2]. 1p36 deletion syndrome represents 0.5–1.2% of total cases of syndromic intellectual disability and it requests multidisciplinary management and intensive follow-up [6] .

Other rare abnormalities associated with 1p36 deletion syndrome include telangiectatic skin lesions and hyperpigmented macules, polydactyly, congenital spinal stenosis, congenital fiber type disproportion myopathy, redundant skin on the nape of the neck, intestinal malrotation, annular pancreas and anomalous arrangement of the pancreaticobiliary duct, liver steatosis, hypertrophic pyloric stenosis, anteriorly placed or imperforate anus, hooked or bilobed gallbladder and small spleen, neuroblastoma (in 3 individuals), pemphigus vulgaris (in 1 individual) [4], cutis laxa [9], biliary atresia [10]. Dental agenesis or other dental anomalies have not been described in previous reports.

## Case presentation

We report on two sisters (6 and 8 years of age) with 1p36 deletion due to a germline mosaicism in one of the parents, presenting with a new phenotypic trait, namely a mild intellectual outcome associated with significant dental anomalies.

The two sisters described in this report are the only daughters of unrelated parents coming from China.

Both the parents are in good health and no cases of intellectual disability, miscarriage or syndromes have been reported in their families. The patients were evaluated in several departments (genetic, child neuropsychiatric, cardiology, odontostomatology, ophthalmology, otorhinolaryngology) of Institute for Maternal and Child Health, IRCCS Burlo Garofolo, Trieste, Italy. They underwent investigations such as electrocardiogram, echocardiogram, dental orthopantomography X-Ray (OPT-XR) and Computed Tomography (CT), electroencephalograms, abdomen ultrasound, blood tests, Intelligence Quotient (IQ) tests, genetic analysis.

**Fig. 1:**
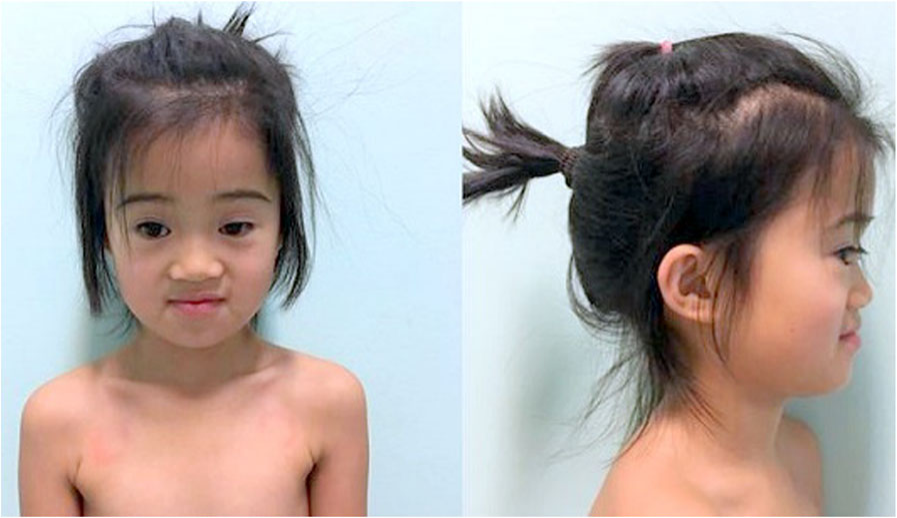
Patient 1. Dysmorphic features: midfacial hypoplasia, bilateral epicanthic fold, upslanting palpebral fissures, palpebral ptosis, nasal bone hypoplasia, dental occlusion of Class III

**Fig. 2:**
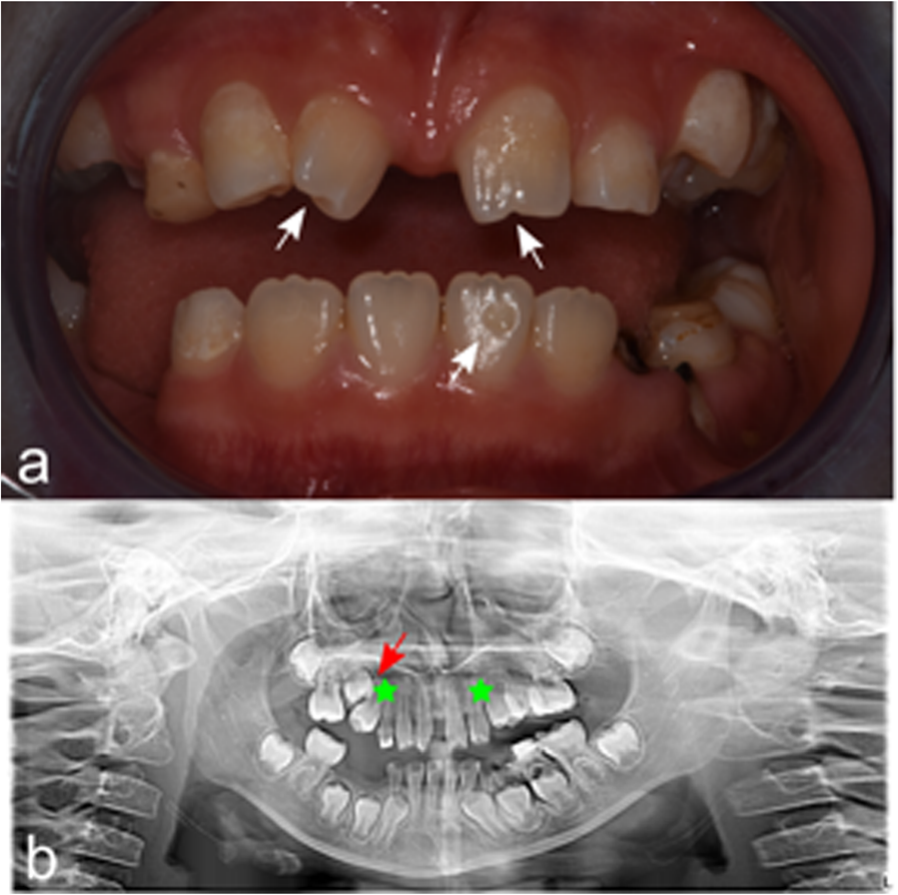
Patient 1. **a** Dental anomalies. Note the atypical shape of the permanent upper incisors (peg- shaped and notched; white arrows) and the areas of enamel demineralization (white and yellow-brown spots diffused and the lack of enamel –pitting-) on the vestibular surface of the inferior central incisor (white arrow). **b** Orthopantomography X-Ray (OPT-XR). The dental agenesis of the two upper canines was identified (canine position is indicated with stars) together with the dystopic eruptive direction (distoangulation) of the still included upper second premolar (red arrow). The germs of the wisdom teeth were not visible

Patient 1 was referred at the age of six for genetic counselling because of suspected learning disorders according to the Diagnostic and Statistical Manual of Mental Disorders, Fifth Edition (DSM-V) [11]. She was born at the 40 GW via spontaneous vaginal delivery following an uncomplicated pregnancy. Ultrasound monitoring was normal and invasive prenatal analysis had not been performed. Birth weight was 3030 g (50-90th centile) [12], birth length was 48 cm (10-50th centile) [12] and birth Occipito Frontal Circumference (OFC) was 34 cm (50-90th centile) [12]. Her neonatal history was unremarkable, including negative hearing screening. She was breastfed for five months and then regularly weaned. Developmental milestones were normal. She sat-up at 6 months of age, walked at 13 months and spoke at 9 months. No history of seizures, fractures or dislocations was reported. Genetic analysis (Single Nucleotide Polymorphism (SNP)–array) identified a deletion 1p36.32 of about 2.3 Mb, typical of the 1p36 deletion syndrome. The patient was clinically evaluated with abdomen ultrasound, blood tests, cardiologic consult with electrocardiogram and echocardiogram that were all negative. Intellectual developmental evaluation at 6-year-old with the Wechsler Preschool and Primary Scale of Intelligence, Third Edition (WPPSI-III) showed a total IQ of 82. Now she attends the primary school full time without any support. She speaks Italian and Chinese and she studies English at school. She was recently evaluated again with the Wechsler Intelligence Scale for Children, Fourth Edition (WISC-IV) reporting an IQ of 75. At the time of the last visit (8 years), her height was 119.8 cm (3–10° centile), her weight was 20 kg (< 3° centile). Physical examination was remarkable for a pronounced ligamentous laxity of elbows and hands (especially of the thumb) with a score of 6/9 at the Beighton scale, hockey-stick palm crease on her left hand, bilateral fifth finger clinodactyly, one hyperpigmented macule on the abdomen and on the left-knee. Neurological examination resulted normal. Dysmorphic features of the patient included midfacial hypoplasia, bilateral epicanthic fold, upslanting palpebral fissures, nasal bone hypoplasia, dental occlusion of Class III (Fig. 1). It was reported that she underwent extractions of two deciduous teeth (inferior right posterior deciduous molars) due to caries; at the time of the last visit, other deciduous teeth (inferior left deciduous canines and molars) were still decayed as well as the residual roots of upper right and left last deciduous molars. The inferior second left deciduous molar was already treated with restoration. Through oral investigation, dental OPT-XR and CT we found dental anomalies of shape, number and position. The clinical crowns of the erupted upper incisors had a diameter smaller than usual; moreover, they were cylindrical, with a horny look and presented with a depression in the middle of the incisal portion (peg- shaped and notched). The dental enamel was affected by areas of demineralization (white and yellow-brown spots), the left central inferior permanent incisor had a lack of enamel (pitting) on the vestibular surface. White and brown spots were diffuse (Fig. 2a). Dental agenesis of the two upper canines was found; the germs of the wisdom teeth were not visible. The first right premolar had a dystopic eruptive direction (distoangulation), and it was still in inclusion (Fig. 2b).

**Fig. 3:**
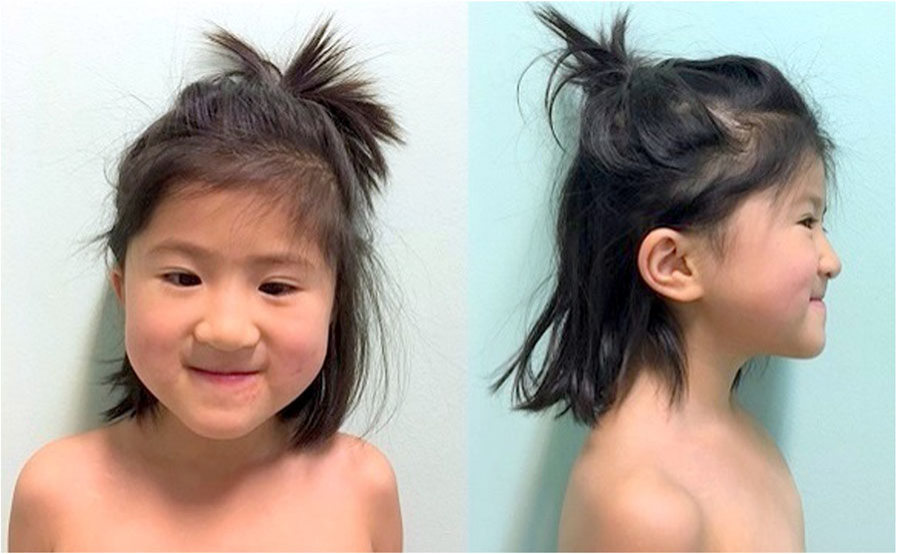
Patient 2. Dysmorphic features: bilateral epicanthus, low-set ears, severe midfacial hypoplasia, flat occiput

**Fig. 4:**
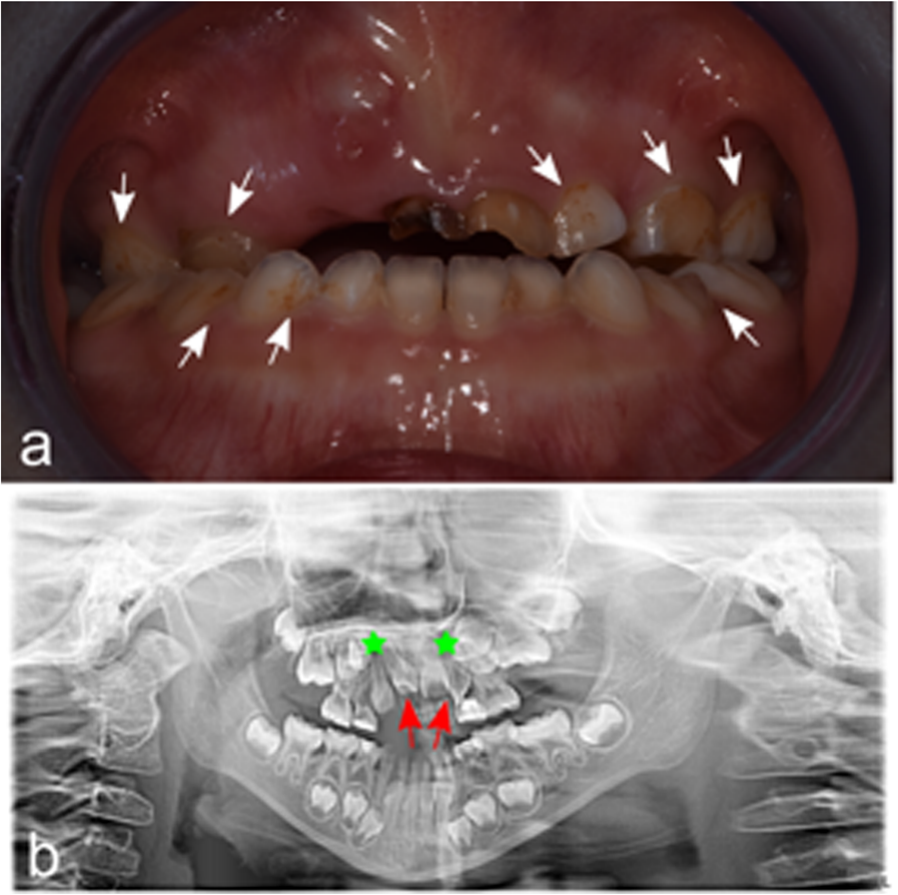
Patient 2. **a** Dental anomalies. Note the diffused demineralization (white and brown spots; white arrows). **b** Orthopantomography X-Ray (OPT-XR). Dental agenesis of the two upper canines was identified (canine position is indicated with stars). The suspected shape anomaly of the upper incisors is indicated with red arrows

Patient 2, the younger sister of Patient 1, was referred for genetic counselling at five years of age, due to a cardiac disease and positive family history for 1p36 deletion syndrome. The baby was born by cesarean section at 39^+ 6^ GW because of breech presentation. Birth weight was 3120 g (10–50th centile) [12], birth length was 48 cm (10-50th centile) [12] and OFC was 36.2 cm (>97th centile) [12]. Newborn hearing screening was normal. Immediately after birth, in order to investigate a systolic murmur, an echocardiogram was performed revealing a patent arterial duct and patent foramen ovale, slight flattening of the interventricular septum, mild tricuspid deficiency and dilated cardiomyopathy with left-ventricular non-compaction. At five months of age, she developed left heart failure (Ejection Fraction = 23%) and started a treatment with metoprolol, digoxin, furosemide, captopril and warfarin. Two months later, she suffered from right frontal subdural hemorrhage with epileptic seizure and she was treated with midazolam and surgical drainage. A history of poor growth due to feeding difficulties, that required a nasogastric tube, and a moderate hypotonia are documented in her medical records. Developmental milestones were almost normal: she walked at 13 months of age and spoke at 12 months. Despite this, since the age of 4 she has been undergoing speech therapy rehabilitation for a mild language delay and she is making gradual progress. She speaks both Italian and Chinese. At 6-year-old she was on treatment with metoprolol, ramipril, levetiracetam and acetylsalicylic acid. Electroencephalograms continue to show epileptogenic anomalies. Genetic analysis (SNP array) identified the same 1p36.32 deletion of the sister. SNP-array does not identify any anomalies in the parents, suggesting a germline mosaicism. Paternity was confirmed. Intellectual developmental evaluation at 6-year-old with the WPPSI-III showed a total IQ of 78. The Otorhinolaryngologist consult indicated a normal bilateral hearing. Eye examination showed strabismus, therefore corrective lenses were prescribed and later surgery was performed. At the time of the last visit (6 years), her height was 107 cm (3–10° centile) and weight was 16 kg (3–10° centile). Physical exam was significant for post-natal microcephaly, ligamentous laxity of the elbows with a score of 2/9 at the Beighton scale. Hockey-stick palm crease on her right hand, bilateral short fifth finger and one hyperpigmented macule on the back were also observed. Dysmorphic features of the patient included bilateral epicanthus, low-set ears, severe midfacial hypoplasia, flat occiput, two posterior hair whorls, high palate (Fig. 3). Neurological exam resulted normal. The odontostomatological examination revealed a severe III Class malocclusion in a micrognathia pattern with a right posterior crossbite, massive caries of the deciduous upper incisors, small caries of both second upper deciduous molars. The first deciduous molars have been already treated with restoration. Areas of demineralization (white and brown spots) are diffused (Fig. 4a). X-Ray and CT examination showed agenesis of the upper canines and malposition of the central right upper incisor and the lateral left upper incisor; following the X-ray report, a shape anomaly of the upper incisors is suspected, though it needs to be confirmed after the eruption (Fig. 4b).

**Fig. 5:**
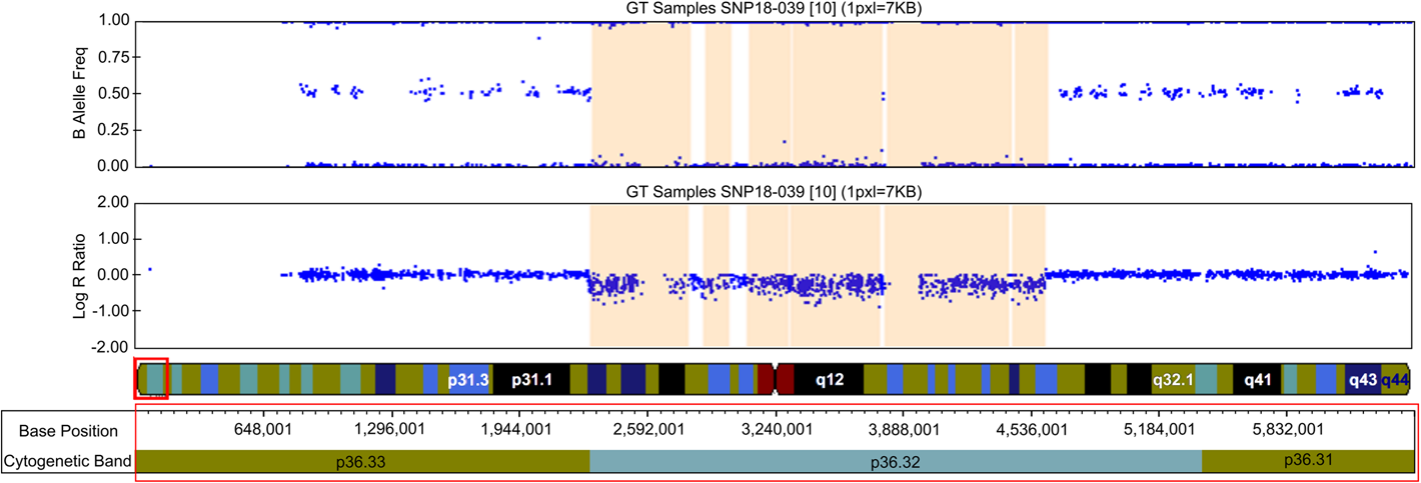
Oligonucleotides array profile of our patients shows 1p36.32 deletion of 2,3 Mb. OMIM genes disease-causing inside the critical region 1p36.32, according UCSC: *PEX10*, *PRDM16*, *CEP104*

SNP–array analysis (ILLUMINA ExpressExome arrays) on blood samples of the two sisters identified a deletion 1p36.32 of about 2.3 Mb (arr [hg19] 1q36.32(2.309.082 × 2, 2.310.193–4.634.978 × 1, 4.636.767 × 2)), typical of the 1p36 deletion syndrome (Fig. 5). This analysis was performed also on the parents, demonstrating that they did not carry any pathogenic microduplication or microdeletion and confirming the paternity. Genotype data of the SNPs located in the deleted region allowed to prove that the 1p36.32 deletion occurred on the maternal allele. Karyotype analysis and Fluorescent In Situ Hybridization analysis (FISH analysis) with probes Vysis LSI P58 and Vysis 1pTEL did not showed subtelomeric rearrangements or chromosomal translocations in the parents (see Additional file 1). Clinical exome sequencing (Clinical Exome Solution-SOPHiA Genetics) was performed on the patient 1 to exclude small deletions/duplications or single nucleotide variants in known genes associating with dental anomalies.

During the evaluation of the patients, the mother informed us she was pregnant and at gestational age of 12 weeks she underwent prenatal Comparative Genomic Hybridization-array (CGH-array) analysis (DNA extracted from chorionic villous sampling). CGH-array was performed by a different laboratory and revealed that the fetus did not carry the 1p36 deletion or other quantitative alterations.

## Discussion and conclusions

To date, no dental anomalies in patients with 1p36 deletion syndrome have been reported in literature. Our patients, however, presented with an uncommon clinical feature, namely the dental anomalies. Interestingly, this characteristic was the only phenotypic feature shared by both sisters. The oral phenotype is well represented and characterized by malocclusion, canine agenesis and dental malposition. The shape anomaly found in the permanent upper incisor is uncommon and it was similar to the *Hutchinson’s incisors* sign of congenital syphilis. Only after their eruption, it will be possible to confirm whether the permanent teeth of the younger sister show the same shape and structural dental anomalies recorded for the oldest sister. Both patients had a severe III Class occlusion, while both their parents had a normal occlusion of Class I. Tooth agenesis is the most common craniofacial malformation in humans [13]. It should be reported that the mother presented the agenesis of the left upper lateral incisor, the most frequent dental agenesis of permanent teeth with the mandibular second premolars [14, 15], while the maxillary canines were missing in our syndromic patients.

Both of the children had a high cario-receptivity despite a good oral hygiene; however, it is not possible to establish whether this is due to the structural anomaly in the deciduous teeth or to sugar-rich diet. The surface with yellow-brown spots of the permanent teeth of patient 1 suggested a diffuse hypomineralization with an increased caries risk.

In the light of the present findings, an early dental examination and an orthodontic visit with a strictly follow up are highly recommended in order to avoid the insurgence of caries. Moreover, an interceptive orthodontic therapy for an initial III Class correction would minimize the future problem of dental agenesis and malposition disturbances in the spacing of the dentition and would allow an adequate nutrition for an appropriate growth and development. Furthermore, even though they are easily recognizable with a simple oral investigation, so many anomalies of shape, number, position and enamel surface associated with a severe III Class are rarely found in clinical practice. Therefore, they could be the cue for further investigation of several organs and genetic analysis.

We searched for OMIM genes included in the 1p36 deletion and we did not find candidate genes that may cause impaired odontogenesis. To exclude the co-segregation of 1p36 deletion with pathogenic variant/s in known genes associating with dental anomalies, we performed clinical exome sequencing on the patient 1. No small deletions/duplications or point mutations were identified in candidate genes (see Additional file 2). Hence, even though we cannot attribute this aspect unquestionably to monosomy 1p36, it is likely that this is an underdiagnosed finding of 1p36 deletion syndrome spectrum.

To date, there are two reports of a recurrent monosomy 1p36 due to probable germline mosaicism. Our data, as well as the findings of Di Donato N et al. (2014) and Gajecka M et al. (2010), confirm that the germline mosaicism seems to be the only explanation for the presence of the same 1p36 deletion in two sisters born from healthy parents [2, 5].

The normal result of CGH-array of the fetus supports the hypothesis of a germline mosaicism. Our findings, as well as Di Donato N et al. (2014), confirm that the recurrence risk for a couple that has a child with a de novo 1p36 deletion might be higher than the general population risk [2] and that germline mosaicisms may be more common than expected.

Our patients present with a very peculiar phenotype. Patient 1 has no cardiac malformations, seizures or language delay, while patient 2 has dilated cardiomyopathy with left-ventricular non-compaction, epilepsy, strabismus, history of poor growth, hypotonia and mild language delay.

Although Patient 1 was referred for suspected learning disorder, she has an IQ of 75 and she attends the primary school full time without any support. The second patient has an IQ of 78 and presents a mild psychomotor delay. It is also significant to consider that the girls speak three languages, Chinese at home and Italian and English in school. We suggest that most of the patients with 1p36 deletion syndrome have been described with severe intellectual disability for selection bias as arrays analysis is performed more often in patients with lower IQs and with severely impaired cognitive function.

Di Donato at al [2] described two brothers with 1p36 deletion syndrome not involving *KCNAB2* and *GABRD* genes and without epileptic history. They, therefore, supposed that haploinsufficiency of one of these genes could cause epilepsy in patients with 1p36 deletion syndrome. However, the deletion found in our probands does not involve *KCNAB2* and *GABRD* genes or other OMIM genes known to be related to epilepsy, but nevertheless patient 2 has an epileptic history. Therefore, we suggest that haploinsufficiency of genes other than *KCNAB2* and *GABRD* may cause seizures in patients with 1p36 deletion with an incomplete penetrance.

The deletion of our patients involves the *PRDM16* gene, previously associated with cardiomyopathy in 1p36 deletion syndrome [16]. The fact that only one of our patients suffers from cardiomyopathy supports the hypothesis that the penetrance of *PRDM16* haploinsufficiency is incomplete and thus the deletion of this gene is not enough to develop cardiomyopathy, as previously reported [2].

In conclusion, we report the third study with recurrent 1p36 deletion syndrome in two siblings, due to a germline mosaicism. Our two patients show very mild intellectual impairment and present with dental anomalies, never described before in association with 1p36 deletion syndrome. This report suggests that 1p36 deletion syndrome may presents also with mild cognitive impairment or even with normal intellectual development, as previously described [2]. This finding is very important for genetic counselling, especially in a prenatal setting. We suggest that 1p36 deletion syndrome may be sometimes underdiagnosed because of the possible absence of cognitive phenotype impairment. Furthermore, we believe that the dental anomalies should be investigated in 1p36 deletion syndrome and that the spectrum of the condition could be broader than we assume.

## Supplementary information

**Additional file 1: Figure 6** - FISH analysis. FISH analysis reveals the presence of 1p36 region in each chromosome 1. The arrow indicates 1p36 region, the orange spot corresponds to *CDK11B* gene and the green spot corresponds to *AGRN* gene. The dotted arrow indicates 1q25 region to demonstrate that this is the chromosome 1. **a**) Father assay; **b**) Mother assay; **c**) Probes used for the analysis: LSI P58 (orange) and Vysis 1pTeL (green) for 1p36 region; LSI 1q25 (aqua) for 1q25 region.

**Additional file 2.** Clinical Exome Sequencing (SOPHiA) of the patient 1. Table summarizes genes analysed and the coverage for each exon. No pathogenic/likely pathogenic variants were identified in candidate genes (WNT10A, MSX1, LRP6, WNT10B, PAX9, EDA, AXIN2, EDARADD, FGFR1, IRF6) or in genes included in the virtual panel generated by SOPHiA Software (SOPHiA-DDM-v4) using the following HPO terms: oligodontia; dental anomalies (virtual panel: ANKRD11; AXIN2; BCOR; CCBE1; CLDN1; EDA; EDARADD; FAT4; KCNJ5; POLR3A; RNU4ATAC; FGFR1; HOXD13; IRF6; KCNJ2; LRP6; LTBP3; MSX1; NAA10; PAX9; POLR1C; POLR3B; PORCN; PTHLH; SATB2; SH3BP2; SLC29A3; TGFA; TP63; WNT10A).

## Data Availability

All data generated or analysed during this study are included in this published article.

## Abbreviations

OFC: Occipito Frontal Circumference
CT: Computed Tomography
MRI: Magnetic Resonance Imaging
FISH: Fluorescent In Situ Hybridization
SNP-array: Single Nucleotide Polymorphism-array
OPT-XR: Orthopantomography X-Ray
IQ: Intelligence Quotient
WPPSI-III: Wechsler Preschool and Primary Scale of Intelligence, Third Edition
CGH-array: Comparative Genomic Hybridization-array
